# Historical overview and new directions in bioarchaeological trace element analysis: a review

**DOI:** 10.1007/s12520-020-01262-4

**Published:** 2021-01-15

**Authors:** Rachel Simpson, David M. L. Cooper, Treena Swanston, Ian Coulthard, Tamara L. Varney

**Affiliations:** 1 Department of Archaeology and Anthropology, University of Saskatchewan, Saskatoon, SK Canada; 2 Present Address: Department of Anthropology, University of Alberta, Edmonton, AB Canada; 3 Department of Anatomy, Physiology and Pharmacology, University of Saskatchewan, Saskatoon, SK Canada; 4 Department of Anthropology, Economics and Political Science, MacEwan University, Edmonton, AB Canada; 5 Department of Biological Sciences, MacEwan University, Edmonton, AB Canada; 6 Canadian Light Source, Saskatoon, SK Canada; 7 Department of Anthropology, Lakehead University, Thunder Bay, ON Canada

**Keywords:** Bioarchaeology, Trace element analysis, Diagenesis, Micro-sampling, Element mapping, Non-traditional stable isotope analysis

## Abstract

Given their strong affinity for the skeleton, trace elements are often stored in bones and teeth long term. Diet, geography, health, disease, social status, activity, and occupation are some factors which may cause differential exposure to, and uptake of, trace elements, theoretically introducing variability in their concentrations and/or ratios in the skeleton. Trace element analysis of bioarchaeological remains has the potential, therefore, to provide rich insights into past human lifeways. This review provides a historical overview of bioarchaeological trace element analysis and comments on the current state of the discipline by highlighting approaches with growing momentum. Popularity for the discipline surged following preliminary studies in the 1960s to 1970s that demonstrated the utility of strontium (Sr) as a dietary indicator. During the 1980s, Sr/Ca ratio and multi-element studies were commonplace in bioarchaeology, linking trace elements with dietary phenomena. Interest in using trace elements for bioarchaeological inferences waned following a period of critiques in the late 1980s to 1990s that argued the discipline failed to account for diagenesis, simplified complex element uptake and regulation processes, and used several unsuitable elements for palaeodietary reconstruction (e.g. those under homeostatic regulation, those without a strong affinity for the skeleton). In the twenty-first century, trace element analyses have been primarily restricted to Sr and lead (Pb) isotope analysis and the study of toxic trace elements, though small pockets of bioarchaeology have continued to analyse multiple elements. Techniques such as micro-sampling, element mapping, and non-traditional stable isotope analysis have provided novel insights which hold the promise of helping to overcome limitations faced by the discipline.

**Supplementary Information:**

The online version contains supplementary material available at 10.1007/s12520-020-01262-4.

## Introduction

Bones and teeth act as reservoirs for several minor and “trace” elements that circulate through the body, and consequently, the skeleton represents a record of one’s lifetime element exposure. Trace element (TE) analysis of human skeletal remains can be useful from a bioarchaeological standpoint because TEs provide rich insights into past human diet, health, status, occupation, and activities. While theoretically valuable for interpreting the past, bioarchaeological TE analysis has previously faced numerous critiques, primarily regarding diagenesis and the reliance upon unsuitable elements. This review will analyse the state of bioarchaeological TE analysis by providing a historical overview of the field, summarizing the current state of the discipline, and highlighting approaches with growing momentum for TE analysis.

Literature included in this review was compiled using general academic search engines (Google Scholar and institutional library databases) and surveying archives of relevant journals corresponding to time periods of interest. Inclusion criteria for the bulk of literature included are TE studies of archaeological bones and teeth, though contributions from modern research are included, where necessary, to contextualize bioarchaeological methods and inferences. One limitation of this review is that the cited literature is almost solely in English. Much TE research has been carried out over the past few decades in languages besides English that are not covered by this review, but as far as we are aware, the basic underlying trends outlined here are consistent with those of the discipline as a whole.

## Background

Bones consist of approximately 60% inorganic mineral, 30% organic matrix, and 10% water (Feng 2009). The organic matrix of bone is primarily comprised of collagen fibres studded with inorganic hydroxyapatite (Ca_10_(PO_4_)_6_(OH)_2_) mineral crystals. Teeth are comprised of three distinct tissues: enamel, dentin, and cementum, which similarly consist of an organic collagenous matrix and inorganic hydroxyapatite crystals. The organic to inorganic ratio varies according to each dental tissue: approximately 96% inorganic constituents and 4% organic constituents in enamel; 70% inorganic constituents, 20% organic constituents, and 10% water in dentin; and 45–55% inorganic constituents and 50–55% organic matrix and water in cementum (Bhaskar 1991; de Dios Teruel et al. 2015).

TEs circulating through the body can become incorporated into actively forming regions of skeletal tissues. In the case of remodelling bone, TE incorporation occurs throughout an individual’s life, as bone undergoes an ongoing natural cycle of turnover consisting of osteoclastic bone resorption followed by osteoblastic bone formation. Initial secretion of organic matrix is followed by the twofold process of mineralization, in which 50–70% of hydroxyapatite crystals are rapidly added during the process of primary mineralization (6 months in ewe [*Ovis aries*] animal model) and the remaining 30–50% continue to be added and gradually mature during the process secondary mineralization (30 months in ewes; estimated to be a couple years in humans; Bala et al. 2010; Ruffoni et al. 2007). Bone turnover rates are tissue and bone specific, with trabecular (cancellous) bone often turning over at a much more rapid rate (e.g. 17.7% per year in the ilium) than cortical bone (e.g. 7.7% per year in the ilium), and ribs and phalanges turning over far more rapidly (e.g. couple years to completely remodel) compared to long bones such as the femur (e.g. years to decades to completely remodel; Frost 1969; Hill 2014; Parfitt 2002; Hedges et al. 2007; Skedros et al. 2013; Fahy et al. 2017). Hedges et al. (2007) used radiocarbon residual in bone from Cold War bomb testing as a tracer to determine bone collagen turnover rates in 67 adults ranging from 40 to 97 years of age at death. They discovered that mean femoral bone collagen turnover rates were lower than previously thought for males (1.5–3% per year) and females (3–4% per year), with bones from some adult individuals still containing collagen that formed during adolescence. It should be noted that while this study used bone collagen, there are no equivalent studies on apatite turnover, and it is anticipated that collagen and apatite turnover rates would be roughly equal due to the indiscriminate process of remodelling. Bone remodelling rates are subject to change across the life course as a consequence of age, with childhood bone turning over much more rapidly than mature adult bone, and age-related conditions such as osteoporosis resulting an imbalance of bone formation vs bone resorption (Szulc et al. 2000; Cheung et al. 2010). Additionally, variables such as mechanical stress, diet, health, sex, ancestry, and lifestyle may also alter the rate of bone remodelling (Carter 1984; Martin and Armelagos 1985; Szulc et al. 2000; Cho et al. 2006; Schulman et al. 2011). In sum, the majority of TEs are incorporated into actively forming regions of bone undergoing primary mineralization, bone as a whole represents a composite mosaic of TE exposure dating back years to decades, and this window of time is reliant on a number of lifestyle, demographic, and health factors.

Barring the surface of the tooth crown, which fluctuates through periods of mineralization and demineralization, sometimes due to interaction with saliva (Abou Neel et al. 2016), teeth do not remodel, and the time sequence of TE incorporation is tissue dependent. Primary dentin, the inner portion of teeth comprising the root and majority of the crown, and enamel, the hard outer covering of the tooth, incrementally form layers during childhood via odontoblast and ameloblast activity, respectively. Secondary dentin begins to gradually form layers once formation of the root is complete (Hillson 1996: 194). By contrast, cementoblast cells continually and incrementally form cementum, the outer layer of the tooth root, throughout one’s lifetime. Therefore, primary dentin and enamel TE composition represent a short-term record of TE exposure, whereas secondary dentin and cementum represent a somewhat linear record of lifetime TE exposure. Different teeth form during established intervals during childhood (Smith 1991; Saunders et al. 1993), so the TE composition of different teeth provide separate windows into specific periods of childhood.

The majority of TEs commonly studied in bioarchaeology (e.g. barium [Ba], copper [Cu], fluorine [F], iron [Fe], mercury [Hg], lead [Pb], magnesium [Mg], manganese [Mn], sodium [Na], strontium [Sr], vanadium [V], and zinc [Zn]) contribute to the inorganic component of bones and teeth, though there are a few exceptions—for example, bromine (Br) and selenium (Se) may preferentially bind to collagen (Brätter et al. 1977). While the calcium (Ca) to phosphate ratio of the hydroxyapatite mineral component of bones and teeth remains relatively fixed (Burton 2008), ions may substitute for different chemical constituents of the hydroxyapatite lattice structure, or alternatively, adsorb onto or react with the surface of the crystal (Neuman and Neuman 1958). Some TEs such Ba, Pb, and Sr have a strong affinity for the inorganic phase of the skeleton; consequently, up to 99% of the total body burden of these elements is contained within bones and teeth, where they remain sequestered for years or a lifetime, respectively (Saltzman et al. 1990; Cabrera et al. 1999; WHO 2001). Elements with divalent cations (+ 2) are capable of substituting for Ca ions in the hydroxyapatite structure of bones and teeth, given their chemical similarities to Ca, while other ions and compounds are capable of substituting for hydroxyl or phosphate groups in hydroxyapatite. Early experimental research demonstrated that some TEs (e.g. Sr) perhaps more readily integrate within the crystalline structure of hydroxyapatite, while others (e.g. Ba) primarily accumulate on the surface of the crystal, requiring further exchange processes to facilitate a more permanent integration (Stark 1968). Elements such as Pb and Zn may also be capable of binding to non-collagenous proteins like osteocalcin or osteopontin that are particularly rich in the cement lines and central canals of bone (Pemmer et al. 2013); this potential protein-mediated mechanism of incorporation would, in principle, extend to teeth as well, though further research is needed. Table 1 provides an overview of several TEs commonly studied in bioarchaeology contexts, along with pertinent characteristics.

**Table 1:** Properties of TEs commonly studied in bioarchaeology with biogenic modes of uptake

	Element	Atomic number	Atomic mass (amu)	Ions	Stable isotopes (radiogenic isotopes)	% of body levels in bone	Mechanism of incorporation	Essential?	Toxic or harmful?
Alkaline earth metals	Barium (Ba)	56	137.3	+ 2	^130^Ba, ^123^Ba, ^134^Ba, ^135^Ba, ^136^Ba, ^137^Ba, ^138^Ba	90%^1^	Substitute for Ca ions in hydroxyapatite	No	Yes and no^a^
	Magnesium (Mg)	12	24.31	+ 2	^24^Mg, ^25^Mg, ^26^Mg	50–60%^2,3^	Minor component of hydroxyapatite; substitute for Ca ions in hydroxyapatite or adsorption onto crystal surface^3^	Yes	No^b^
	Strontium (Sr)	38	87.62	+ 2	^81^Sr, ^84^Sr, ^86^Sr, ^88^Sr, (^87^Sr)	99%^4^	Substitute for Ca ions in hydroxyapatite	No	No
Transition metals	Copper (Cu)	29	63.55	+ 2	^63^Cu, ^65^Cu	Unknown	Collagen cross-linking function^9^; binds to hydroxyapatite^10^	Yes	No^b^
	Iron (Fe)	26	55.85	+ 3, + 2	^54^Fe, ^56^Fe, ^57^Fe, ^58^Fe	Unknown	Collagen synthesis cofactor^11^; substitutes for Ca ions in hydroxyapatite	Yes	No^b^
	Mercury (Hg)	80	200.6	+ 2, + 1	^198^Hg,^199^Hg, ^200^Hg,^201^Hg, ^202^Hg, ^204^Hg	Unknown	Substitute for Ca ions in hydroxyapatite	No	Yes
	Manganese (Mn)	25	52.94	+ 7, + 4, + 2	^55^Mn	43%^5^	Role in regulating bone remodelling^12^; substitute for Ca ions in hydroxyapatite	Yes	No^b^
	Vanadium (V)	23	50.94	+ 5, + 4, + 3	^51^V	Unknown	Incorporates into hydroxyapatite framework^13^	No	No^b^
	Zinc (Zn)	30	65.39	+2	^64^Zn, ^66^Zn, ^67^Zn, ^68^Zn, ^70^Zn	28%^6^	Metallothionein co-factor^14^; attachment to non-collagenous proteins^15^; substitute for Ca ions in hydroxyapatite	Yes	No^b^
Basic metals	Lead (Pb)	82	207.2	+ 2	^204^Pb, ^206^Pb, (^207^Pb), (^208^Pb)	78–96%^7^	Possibly substitutes for Ca ions in hydroxyapatite though mechanism thought to be more complex than simple substitution^3^; attachment to non-collagenous proteins?^15^	No	Yes
Metalloids	Arsenic (As)	33	74.92	+ 5, + 3	^75^As	Unknown	Arsenate (AsO_4_^3−^) can substitute for phosphate ions in hydroxyapatite^16^	No	Yes
Non-metals	Fluorine (F)	9	18.90	− 1	^19^F	~ 99%^8^	Ions substitute into hydroxyapatite hydroxyl group^17^	No	No ^b^
	Selenium (Se)	34	78.96	− 2	^74^Se, ^76^Se, ^77^Se, ^78^Se, ^80^Se	Unknown	Selenite (SeO_3_^2−^) can substitute for phosphate in hydroxyapatite^18^; association of Se with skeletal collagen^19^	Yes	No
	Bromine	35	79.90	− 1	^79^Br, ^81^Br	Unknown	Association of Br with skeletal collagen^19^	Yes	Yes and no^c^

^1^WHO 2001; ^2^Jahnen-Dechent and Ketteler 2012; ^3^Neuman and Neuman 1958; ^4^Cabrera et al. 1999; ^5^O’Neal et al. 2014; ^6^Hambidge et al. 1986; ^7^Saltzman et al. 1990; ^8^Whitford 1994, p. 5; ^9^Opsahl et al. 1982; ^10^Laurencin et al. 2011; ^11^Tuderman et al. 1977; ^12^Strause et al. 1987; ^13^Petit et al. 2017; ^14^Hoadley et al. 1988; ^15^Pemmer et al. 2013, p. 188; ^16^Dani 2013, p. 542; ^17^Murugan et al. 2002, 429; ^18^Monteil-Rivera et al. 2000; ^19^Brätter et al. 1977

^a^Barium (Ba) not toxic at environmental levels; certain compounds (e.g. BaCl) may be toxic

^b^Low levels not toxic; toxicity at extremely high levels of intake

^c^Some gaseous or liquid forms of bromine (Br) may be toxic at certain levels

## Early trace element analysis of the human skeleton (1950s to mid-1970s)

Mid-twentieth-century clinical and archaeological efforts of chemical bone and dental analysis aimed to define (1) the TEs present in bones and teeth, (2) which of these TEs are essential, and (3) the normal and abnormal levels for each element (Drea 1935; Hodges et al. 1950; Fore and Morton 1952; Brudevold and Steadman 1955; Sowden and Stitch 1957; Steadman et al. 1958; Taylor 1959; Brudevold et al. 1963). Medically, many research questions were also dictated by societal concerns for the radioactive impacts of Cold War nuclear testing on human health. As such, Sr and Ba were of primary interest, given their potential radioactive forms. Among modern individuals from undefined populations, Hodges et al. (1950) and Sowden and Stitch (1957) both found what they deemed relatively consistent intra-individual levels of Sr throughout the postcranial skeleton, and a positive relationship between Sr content and age (e.g. 64–75 μg/g in an infant; 135–180 μg/g among an individual 78 years of age). Ba levels (4–11.4 μg/g) were lower than Sr levels (53.4–146 μg/g), demonstrating a preferential affinity of Sr for bone (Sowden and Stitch 1957). Investigations into Sr and Ba concentrations in teeth revealed intra-enamel and intra-dentin variability and geographic variation among modern individuals originating from Texas, South Dakota, Maine, New Mexico, USA (25–350 μg/g), Greenland (260–300 μg/g Sr), and the islands of Tonga (320–600 μg/g Sr) and Nauru (190 μg/g Sr), as well as in a 5000-year-old archaeological individual from Kentucky (195–300 μg/g Sr; Steadman et al. 1958).

Chemical analyses of archaeological human remains were previously concerned mainly with investigating the permeation of TEs from the soil into ancient bones and teeth (Steadman et al. 1959), and by extension, the potential for chronologically dating remains through analysis of their chemical composition (Heizer and Cook 1952; Cook and Heizer 1953). Oakley (1969, 1980) similarly proposed a method of relative dating of fossilized skeletal remains through assessing the concentrations of fluorine, uranium, and nitrogen (FUN). This method was based on the assumption that fluorine and uranium accumulate in fossilized bones, teeth, and antlers just as steadily as nitrogen content decreases. Early investigations also targeted the distribution of TEs in the bone, using neutron activation analysis to examine diffusion gradients of elements into, and out of, fossil bone, across both exterior and interior bone surfaces (Farquhar et al. 1978; Badone and Farquhar 1982).

It was not until the mid-1960s, however, that TE analysis of archaeological or fossil skeletal remains was used to infer past behaviour. Toots and Voorhies (1965) were the first to apply Sr to Ca (Sr/Ca) ratios in fossil bone to make palaeodietary inferences. Their study was based on Odum’s (1957) finding that mammalian metabolisms follow a Ca biopurification process, in which gastrointestinally, Sr is selectively discriminated against and Ca is preferentially taken up; therefore, Sr/Ca ratios markedly decrease with each increase in trophic level. Toots and Voorhies (1965) proposed that fossil animals’ diets and trophic positions could then be inferred through analysis of the Sr/Ca ratio; carnivores would have a low Sr/Ca ratio when compared with herbivores, and herbivore Sr/Ca ratios would vary according to the type of vegetation typically consumed by a species (i.e. leafy greens are high in Sr while grasses are low in Sr). Similar to Sr, dietary Ba and Pb also follow a Ca biopurification process, and therefore, Ba/Ca and Pb/Ca ratios also decrease with each increasing trophic position (Elias et al. 1982), though while Pb is abundantly taken up into body tissues via contaminated food and drink, it has a number of additional pathways of entry, such as through inhalation or skin (Schroeder and Tipton 1968).

Antoinette Brown’s (1973, 1974) doctoral research constituted the first application of TE analysis of Sr in human skeletal remains to reconstruct palaeodiet. Brown analysed bulk Sr levels in skeletal samples from the Huitzo village site in Oaxaca, Mexico, arguing that differences in Sr concentrations related to differences in plant and meat consumption and that, consequently, social stratification can be inferred by individuals’ differential access to meat protein. This concept was expanded in Robert Gilbert’s (1975) doctoral dissertation, which proposed the measurement of Cu, Mg, Mn, Sr, and Zn to reconstruct palaeodiet. According to Gilbert, low Zn levels represent primarily plant consumption and high Zn levels represent high meat consumption, though he suggested that low Zn levels may also be indicative of high cereal and grain consumption, because the phytate compound in these plants may interfere with Zn absorption. In a similar vein, Wessen et al. (1978) proposed the use of Ba as a determinant of the animal origin of bone artifacts, finding significant differences in seal and terrestrial animal Ba content.

Early bioarchaeological TE analyses also extended beyond palaeodiet. Jarcho (1965) first measured Pb in bone samples from two pre-Columbian sites in Arizona: Kinishba, where Pb-glazed pottery was known to be manufactured, and Point of Pines, where it was not. He found no significant differences in Pb concentrations between bones from Kinishba and Point of Pines, though it is impossible to determine the manufacturers and users of Pb-glazed pottery from the remains at Kinishba. These early efforts of using TEs to recreate past lifeways set the stage for a new era of chemical analysis in bioarchaeology.

## Peak of trace element analysis (mid-1970s to 1990s)

### Trace elements and palaeodiet

Beginning in the late 1970s and extending into the 1990s, the popularity of bioarchaeological chemistry of skeletal remains exploded. Improvements in chemical analytical methods allowed for a wider range of elements to be detected (Price et al. 1985) as well as improved element detection limits. Following Brown’s (1973, 1974) application of Sr and Gilbert’s (1975) proposed use of Cu, Mg, Mn, Sr, and Zn for palaeodietary reconstruction, numerous TEs were used to infer palaeodiet. Sr was considered a staple of TE palaeodietary reconstruction and was used to infer dietary constituents, diachronic changes in diet, and sex- and status-related differences in diet. Schoeninger (1979) used Sr concentrations to infer social status in an ancient population from Chalcatzingo, Mexico (1150–550 BCE), reporting that high status individuals (associated with jade funerary objects) had low bone Sr indicative of high meat consumption, while low status individuals (not associated with grave goods) had high bone Sr indicative of high plant consumption.

Sr concentrations and Sr/Ca ratios became popular in establishing diachronic subsistence shifts—for example, the introduction and increased reliance upon cultivated plants. As iterated above, Sr is impacted by the biopurification of Ca among most mammals and therefore is potentially indicative of trophic level. Price and Kavanagh (1982) used Sr/Ca ratios to examine diachronic changes in diet, arguing that there were increases in plant consumption between the Late Archaic, Middle Woodland, and Mississippian periods throughout present-day Wisconsin. Bone Sr levels were also used in conjunction with stable carbon (δ^13^C) and nitrogen (δ^15^N) isotopes to infer changes in plant consumption (e.g. the possible introduction of maize; Katzenberg 1984; Katzenberg and Schwarcz 1986).

While initially high Sr concentrations or Sr/Ca ratios were thought to be exclusively indicative of plant consumption, later studies demonstrated this was not as simple as initially proposed. Within plants, leaves are higher in Sr than stems, and because certain plants like maize and squash are notably depleted in Sr, relatively low bone Sr levels may also be indicative of maize or squash horticulture (Katzenberg 1984). Consumption of other low-Sr or Ca-rich foods may further complicate Sr/Ca ratios; Schoeninger and Peebles (1981) found that mollusc consumption obscured Sr evidence of high plant consumption and caused ancient agriculturists to have unexpectedly low Sr.

During the 1980s, multi-element analysis was extremely popular in TE analyses of archaeological bone (e.g. Katzenberg 1984; Beck 1985; Hatch and Geidel 1985; Byrne and Parris 1987; Francalacci 1989; İşcan et al. 1989; White and Schwarcz 1989; Arrhenius 1990; Liden 1990). Early practitioners examined correlations and relationships between different element concentrations and ratios, leading to several elements being proposed for use in palaeodiet reconstruction studies. In addition to the continued widespread use of Sr as an indicator of trophic level, Ba, Cu, Mg, Mn, V, and Zn were used to make a variety of palaeodiet inferences.

A popular application of some elements was to establish trophic level. Following a similar biopurification process to Sr (Elias et al. 1982), Ba/Ca ratios in principle reflect trophic level, and by extension, plant versus meat consumption, though Ba was studied to a lesser extent than Sr. Scholars also employed Ba/Sr ratios as an indicator of marine food consumption, due to seawater’s low Ba levels (Burton and Price 1990; Gilbert et al. 1994). Some researchers proposed that Zn enrichment occurs with each increasing trophic level, due to the naturally high levels of Zn in blood and soft tissue (Gilbert 1975; Rheingold et al. 1983). As such, high Zn values were interpreted as an indicator of high meat consumption, and low levels an indicator of high plant consumption. Beck (1985) used Sr and Zn concentrations to attempt to classify populations with hunter–gatherer, agricultural, and horticultural subsistence strategies. Cu was also proposed as an indicator of meat consumption (Schroeder et al. 1966). Arrhenius (1990) argued that Cu levels could also reflect arthropod, specifically maggot, consumption, due to a Cu haemocyte in insects. In her study of Cu, Se, and Sr concentrations in Scandinavian hunter–gatherer and medieval bone samples, Arrhenius (1990) argued that the elevated Cu levels in the hunter–gatherer populations reflect a subsistence strategy heavily weighted in gastropods, molluscs, and arthropods and hypothesized that these populations intentionally grew maggots for subsistence.

Scholars also proposed numerous TEs for interpreting nut consumption. First proposed by Gilbert (1975), Mg was used in palaeodiet reconstruction as an indicator of nut consumption. According to Hatch and Geidel (1985), Mn and V, like Sr, are indicators of plant consumption, but nuts are low in V; therefore, vegetarian and meat-rich diets with or without nuts could potentially be differentiated on the basis of V in conjunction with Cu, Mn, Sr, and Zn levels. Mn was later interpreted as an indicator of diagenetic contamination (Francalacci 1989), due to its very low naturally biogenic levels in humans.

When compared to the wealth of literature on bone, the study of TEs in archaeological teeth for palaeodietary purposes was almost non-existent during this period, with few exceptions (e.g. Kuhnlein and Calloway 1977; Attramadal and Jonsen 1978; Kyle 1986). It is not clear whether this was a conscious choice by researchers to investigate adult palaeodiets or unconsciously driven by previous precedents in the literature.

### Toxic trace element exposure

While most TE analysis studies in bioarchaeology focused on palaeodiet and palaeoecology, toxic TEs were, to a lesser extent, also analysed to make inferences about population exposure. Pb concentrations can inform our understanding of populational Pb use, health, industry, occupation, and social status, as well as differentiate commingled remains or distinguish ancient from modern skeletal material (Aufderheide et al. 1988). Several early efforts of Pb analysis in ancient populations were driven by research questions of how abnormally elevated modern Pb exposure was relative to physiologically “natural” human levels. “Natural” bulk Pb concentrations in both the bones and teeth of early populations ranged from 0.04 to 3.4 μg/g (Ericson et al. 1979; Grandjean et al. 1979; Jaworowski et al. 1985; Grandjean and Jørgensen 1990) and Pb/Ca ratios of ancient Peruvians were similarly found to be one hundredth of the levels observed in modern England and USA (Ericson et al. 1979).

Since the late 1970s, scholars have used bioarchaeological TE analysis to track the history of human Pb exploitation, demonstrating that there was a high pre-industrial peak of Pb production during antiquity (Wittmers et al. 2002). Specifically, Pb was widely exploited by—and even proposed as a contributor to the downfall of—the Roman Empire (Gilfillan 1965; Nriagu 1983) and consequently, Pb of Roman and post-Roman skeletal material has been the subject of much scrutiny (e.g. Mackie et al. 1975; Ahlgren et al. 1980; Molleson et al. 1986; Vuorinen et al. 1990; Aufderheide et al. 1992).

The 1980s saw an increasing focus on the social determinants of Pb exposure (Aufderheide et al. 1981, 1985; Handler et al. 1986; Corruccini et al. 1987). Did certain social groups have differential exposure to Pb? Variation in Pb exposure within a population can be due to a number of social factors, such as habitual activities or access to luxury goods. Aufderheide et al. (1981) argued that plantation owners and enslaved individuals from a colonial Virginia cemetery could be differentiated based on bone Pb concentrations, because plantation owners had elevated Pb exposure from consuming food and drink from luxury pewter dinnerware. Exceptions to this pattern came in the form of a white individual with low bone Pb concentrations interred alongside the Black enslaved individuals, who likely had similar living conditions, and a Black female with abnormally high Pb concentration (96 μg/g). The authors interpret this individual’s high values as work as a housemaid (Aufderheide et al. 1981).

First proposed by Waldron (1981), Pb isotopes can be used to investigate the material source of Pb exposure, due to regional geological variability in Pb isotopic signatures. For example, Reinhard and Ghazi (1992) used Pb isotopes to infer the source of exposure in an ancient Omaha population. Ultimately, they argued that the primary source of Pb exposure originated from Pb- and cinnabar-based cosmetic pigments applied to the body during mortuary rituals (Ghazi et al. 1994). Pb isotopes were also used to infer mobility; among individuals interred in the Roman cemetery at Poundbury Camp, Dorset, the Pb isotopic composition in most individuals was consistent with a British origin, though one child’s Pb isotope signature was identical to Pb ores from Laurion, Greece (Molleson et al. 1986). So similar were these bone and ore Pb values that the authors argued the child immigrated from Laurion, rather than simply consuming imported food and drink from the region (Molleson et al. 1986).

## Critiques of trace element analysis (late 1980s–1990s)

During the late 1980s and 1990s, several scholars raised critiques in the field of TE analysis, particularly regarding (1) erroneous or simplified assumptions on the effects of diagenesis on human remains (Radosevich 1993), (2) the simplification of complex element uptake processes (Radosevich 1993), and (3) the use of elements inappropriate for palaeodietary reconstruction (Klepinger 1990; Ezzo 1994). Additionally, the archaeological TE literature of the time consistently ignored foundational and seminal literature on the exchangeability of ions with apatite (e.g. Neuman and Neuman 1958; Stark 1968), which may have proved useful in understanding the incorporation and utility of different TEs for bioarchaeological investigation.

The process of diagenesis is the suite of post-mortem physical, chemical, and biological alterations to skeletal remains occurring in the mortuary environment (Hedges 2002). With regard to the impact of diagenesis on the TE composition of skeletal remains, the inorganic phases of bone and teeth are of primary interest, because most TEs interact with hydroxyapatite. Groundwater ions, particles, and organic compounds in the depositional environment can adsorb onto the exterior surface of bones or teeth or enter via surface diffusion, pores, or cracks in bones or dental tissues. Here, ionic substitution, ionic exchange, hydroxyapatite recrystallization, or precipitation of new phases may take place (Hedges and Millard 1995; Dudás et al. 2016). Conversely, biogenic elements from the bones and teeth can leach into the mortuary environment (Price et al. 1992). Microbial activity can also diagenetically impact the chemical composition of remains; soil fungi and bacteria and/or intrinsic gut bacteria can physically degrade the integrity of skeletal remains via tunnelling and can alter the chemical composition of bone by introducing elements from the exterior soil or by depositing chemical metabolic byproducts (Grupe and Piepenbrink 1988, 1989; White and Booth 2014).

The extent of diagenesis can be exacerbated by high temperatures (Von Endt and Ortner 1984), acidic soil pH (Gordon and Buikstra 1981), moist and humic soils (Krajcarz 2019), and poor site drainage and hydrological movements (Hedges and Millard 1995; Nielsen-Marsh and Hedges 2000), as well as a myriad of other biological, chemical, and physical site taphonomic factors (Nielsen-Marsh et al. 2007). With regard to bones, small bones are more susceptible to degradation, leading to an underrepresentation of small fauna and infants in the bioarchaeological record (Von Endt and Ortner 1984; Buckberry 2000). Bone pore structure—namely, the size, volume, shape, and distribution of bone pores—also impacts the pervasiveness of diagenesis (Nielsen-Marsh and Hedges 2000) and is one factor contributing to the underrepresentation of osteoporosis in the bioarchaeological record (Bartosiewicz 2008). The physical and/or microbial breakdown of bones and teeth and consequent alteration of pore structure can further increase susceptibility to diagenesis, as heavy metals in the mortuary environment can enter the skeletal tissues through cracks and pores (Hedges and Millard 1995; Rasmussen et al. 2019). Due to this multiplicity of factors, diagenesis does not behave in a linear or predictable manner in skeletal remains (Klepinger et al. 1986).

While diagenesis was not really on the radar of the earliest practitioners of bioarchaeological TE analysis, it became a very critical topic of discussion for scholars in the heyday of TE analysis. However, many such scholars “explained away” the problem of diagenesis by arguing that (1) certain TEs and skeletal tissues were resistant to diagenesis, (2) diagenesis could be identified by the presence of certain elements and alterations to the crystallinity index of hydroxyapatite, or (3) diagenetic contamination could be removed from skeletal samples.

Some scholars proposed that Sr and Zn are resistant to diagenesis (Gilbert 1975; Schoeninger 1979). This assumption was rejected by Sillen (1981), who found a homogenization effect in Sr concentrations of fossil remains at the Hayonim Cave, potentially indicative of diagenesis. Nonetheless, this assumption persevered into the 1980s (e.g. Lambert et al. 1984, 1985). Pb was also previously thought to be immobile in soil if the soil pH was basic (Zimdahl and Skogerboe 1977); however, Waldron (1981) found extremely high levels of Pb (10,228 μg/g) in bone samples from Pb-lined coffins and Pb-contaminated soils, and Wittmers et al. (2008) found that diagenetic Pb contamination had completely obscured the biogenic Pb signal in a First African Baptist Church population (Philadelphia, PA).

Some critics (Pike and Richards 2002; Millard 2006) have pointed out that despite abundant literature on physiologically feasible ranges for most TEs, some studies have continued to infer biogenic elemental exposure from exorbitantly high TE concentrations. For example, Oakberg et al. (2000) reported As concentrations of 1–8.41 ppm as evidence of ancient Cu smelting, when modern TE concentrations from occupationally exposed individuals range from 6 to 210 ppb (Pike and Richards 2002). Similarly, Millard (2006) commented on the implausibility of biogenic bone Pb levels reaching 1139 ppm or Fe levels reaching 21,000 ppm, as reported in Martínez-García et al. (2005). It is therefore critical to consider element concentrations and isotopic ratios from archaeological remains against the backdrop of existing physiological and toxicological research.

While it has been clearly demonstrated that virtually any TE can be affected by diagenesis, scholars have also effectively demonstrated that some skeletal tissues are more resistant to diagenesis than others. For example, cortical bone is relatively less affected by diagenesis than the extremely porous trabecular portion of bone (Price et al. 1992). Due to its small pores and tightly packed structure, tooth enamel is more resistant to diagenesis than bone and dentin (Parker and Toots 1970; Kyle 1986; Budd et al. 2000; Montgomery et al. 2010; Dudás et al. 2016). For this reason, enamel is often used as an indicator of biogenic TE composition while other skeletal tissues (bone, dentin) may be used as an indicator of the diagenetic signal or to help predict the extent of diagenetic alteration of enamel (e.g. Scheeres et al. 2013; Hollund et al. 2015). Cementum is also more susceptible to diagenetic alteration than enamel and it has previously been demonstrated that diagenesis is capable of mimicking its seasonal increment patterns (Stutz 2002).

On a related note, other scholars have argued that certain elements in bone are unlikely to have a biogenic origin and therefore are indicative of probable diagenesis. For example, Lambert et al. (1984, 1985) suggested that aluminium (Al), Ba, Cu, Fe, Mn, potassium (K), uranium (U), and V are likely to diagenetically contaminate skeletal remains, whereas Ca and Na are susceptible to leaching out of skeletal remains. Katzenberg (1984) argued that rare earth elements (REEs) such as zirconium (Zr) and yttrium (Y), and Ca/P ratios are indicative of diagenesis; Zr and Y are unlikely to be biogenically incorporated into the skeleton; and an abnormally high Ca/P ratio is indicative of carbonate contamination. Evaluating REEs and Ca/P ratios continues to be a popular means of identifying possible diagenetic contamination in skeletal remains today (e.g. Willmes et al. 2016; Giffin et al. 2017; Özdemir et al. 2017; Kamenov et al. 2018).

Comparing element concentrations in archaeological skeletal remains and in situ soils is one method used to attempt to identify diagenesis; Lambert et al. (1979, 1984) and Nelson and Sauer (1984) argued that if the concentrations of an element in the soil and remains were inconsistent, then diagenesis did not take place. However, diagenetic activity is not this simple. Bone and teeth “seeking” elements do not often behave according to a simple concentration gradient between the skeletal remains and soil; rather, skeletal remains may disproportionately accumulate certain TEs that have a strong affinity for the inorganic phase of bones and teeth. In fact, the bone is often used as a medium to extract heavy metals from water and soil (Hodson et al. 2001; Chen et al. 2008). According to Pate and Hutton (1988), a simple skeleton–soil comparative approach also fails to consider the quantity of soluble and exchangeable ions actually available in the soil and under what conditions. Element-specific partition coefficients between soil, groundwater, and skeletal remains need to be considered (Pike and Richards 2002). For example, the high partitioning for U between groundwater and bone can result in excessively higher diagenetic U concentrations in bone relative to typical biogenic levels and soil levels (Millard and Hedges 1995). In sum, while there is value in extracting soil samples for the study of diagenesis, the intricacies of skeleton–soil dynamics and element-specific interactions need to be considered.

Another approach to identify the presence of diagenesis in bones and teeth is to examine the crystalline integrity of remains, through methods such as Fourier transform infrared spectroscopy (FTIR) or Raman spectroscopy. Hydroxyapatite crystal perfection is a sign that diagenesis has increased crystallinity and formed larger, more regular crystals, and as such, various crystallinity indexes have been proposed to aid in assessing the extent of diagenesis (Shemesh 1990; Person et al. 1995). While crystallinity values may inform early diagenetic alterations to skeletal remains, they are less reliable for long-term diagenetic changes, which are often unpredictable and site dependent (Trueman et al. 2008).

It has been consistently demonstrated that diagenetic contamination is often most concentrated at the subperiosteal and endosteal surfaces of bone (Price et al. 1992; Wittmers et al. 2008; Rasmussen et al. 2019). Price et al. (1992) reviewed and evaluated the efficacy of methods such as mechanical cleaning, chemical cleaning, and washing with a reducing agent for removing diagenetic alteration. They found that the success of such methods depends on both the extent of diagenetic contamination and the element in question. Leaching bone samples in a weak acid has been shown to remove some diagenetic Sr, though this can only be said for Sr occupying pores in bone; if Sr has pervaded the bone and become incorporated into the hydroxyapatite crystalline structure of the bone, then an acidic soak is unable to remove it (Beard and Johnson 2000). A weak acid treatment has also been shown to be quite effective at removing diagenetic Sr from enamel pore spaces (Hoppe et al. 2003).

A second critique of TE analysis during the late 1980s and 1990s was the widespread simplification of complex element uptake processes for palaeodietary inferences. For example, it has been demonstrated that Sr not only can widely vary in response to dietary factors but also is subject to vary in response to local geological fluctuations, demographic variables, and culinary practices (Katzenberg et al. 2000) and the Sr/Ca ratio can be swayed by high Ca foods and minor dietary contributions (Burton and Wright 1995), becoming convoluted in mixed, omnivorous diets. It was previously assumed that Sr/Ca ratios reflect trophic level and that humans following omnivorous diets would have Sr/Ca ratios midway between herbivore and carnivore values. However, Runia (1987) showed that some plants may discriminate between Sr and Ca more than initially thought. Furthermore, the bioavailability and mobility of Sr for uptake into plants varies according to regional microbial ecology and soil condition factors, such as pH and temperature (Burger and Lichtscheidl 2019). Sr values also vary according to an animal or human’s sex and age (Sillen 1988), with notable Sr elevation occurring in pregnant and lactating females (Price et al. 1986; Blakely 1989). In sum, Sr/Ca ratios do not behave linearly and reflect a complexity of factors beyond plant contribution and trophic level.

A third major critique of the field of TE analysis was an overzealous optimism for using certain TEs for palaeodietary reconstruction, despite a lack of a scientific justification. According to Joseph Ezzo (1994), for an element to carry any palaeodietary significance, it must satisfy the following conditions. First, the element must have a mode of biogenic incorporation into the skeleton and ideally, the majority of the body burden for that element should be in the skeleton. Second, levels in the skeleton must relate in some way to the levels in diet. Third, the element must not be under homoeostatic control. Several TEs used in the era of multi-element palaeodiet reconstruction do not satisfy these conditions; many of the commonly studied TEs have crucial biological functions and are therefore homoeostatically regulated. Klepinger (1990) experimentally demonstrated this by feeding two groups of pigs (*Sus* sp.) identical diets with high and low Mg supplementation, respectively. Despite one group consuming almost twice the amount of Mg as the other, there were no significant differences in bone Mg levels between the two groups, demonstrating the effect of homoeostatic regulation on Mg metabolism.

In his critique of the widespread use of Zn in TE analysis, Ezzo (1994) argued that unlike Sr, there was currently insufficient medical and scientific evidence that skeletal Zn levels are related to certain dietary components or to trophic level, yet a general uncritical acceptance for Zn’s palaeodietary utility exists among many bioarchaeologists. Zn is known to be a metalloenzyme cofactor that is under homoeostatic regulation (Hoadley et al. 1988; King et al. 2000) and the skeleton’s Zn stores represent an estimated 28% of the entire body burden of Zn (Hambidge et al. 1986). To further complicate the matter, virtually all bioarchaeological studies implementing Zn concentrations for palaeodietary inferences cited the same few works in the literature (i.e. Gilbert 1975, 1977; Lambert et al. 1979, 1982; Blakely and Beck 1981; Hatch and Geidel 1985; Beck 1985) as their scientific rationale. The majority of these cited works treated correlational multi-element data in archaeological remains as empirical evidence, rather than drawing upon the physiological literature or controlled scientific studies (Ezzo 1994). Ezzo emphasized the need for further empirical and controlled biomedical, physiological, ecological, and environmental studies to be undertaken before concentrations of Zn and other essential TEs could feasibly be applied to palaeodietary reconstruction. Following this period of critiques, TE analysis of bioarchaeological remains for palaeodietary reconstruction largely fell out of favour. Since then, stable isotope analysis of bone has primarily been the focus of archaeological bone chemistry, though Sr and toxic TEs continue to be an exception.

## Post-critique: trace element analysis in the twenty-first century

### Trace element isotopes for the study of migration and mobility

Following the abundant critiques of bioarchaeological TE analysis, there has been a shift toward employing TEs strictly for mobility and element exposure studies, commonly through evaluating the radiogenic isotope ratios of Sr and Pb. Because ^87^Sr/^86^Sr isotope analysis of archaeological skeletal remains has been the subject of numerous other reviews (e.g. Bentley 2006; Szostek et al. 2015; Burton and Katzenberg 2018), it will only be briefly reviewed below. Of the four Sr isotopes present in bedrock, ^87^Sr is a radiogenic product of the decay of ^87^Rb, whereas ^86^Sr is a stable, non-radiogenic Sr isotope. ^87^Sr/^86^Sr isotopes in bones and teeth originate from the food and water consumed by the individual, which in turn, reflect the Rb to Sr isotopic composition of the local bedrock. Weathering of rocks releases Sr sequestered in bedrock into the local stream water, atmosphere, and soils, where it can be incorporated into plants and consumed by living things (Bentley 2006). “Isoscapes” based on regional geological ^87^Sr/^86^Sr variability in bedrock and water systems can be constructed to infer provenance (Bataille and Bowen 2012). Consequently, in assessing ^87^Sr/^86^Sr composition of bioarchaeological remains, there is the potential for interpreting mobility and migration in past populations and, by extension, nomadic or migratory behaviour, subsistence strategies, geographic source of imported food or drink, social residence patterns, and sociopolitical dynamics.

**Fig. 1:**
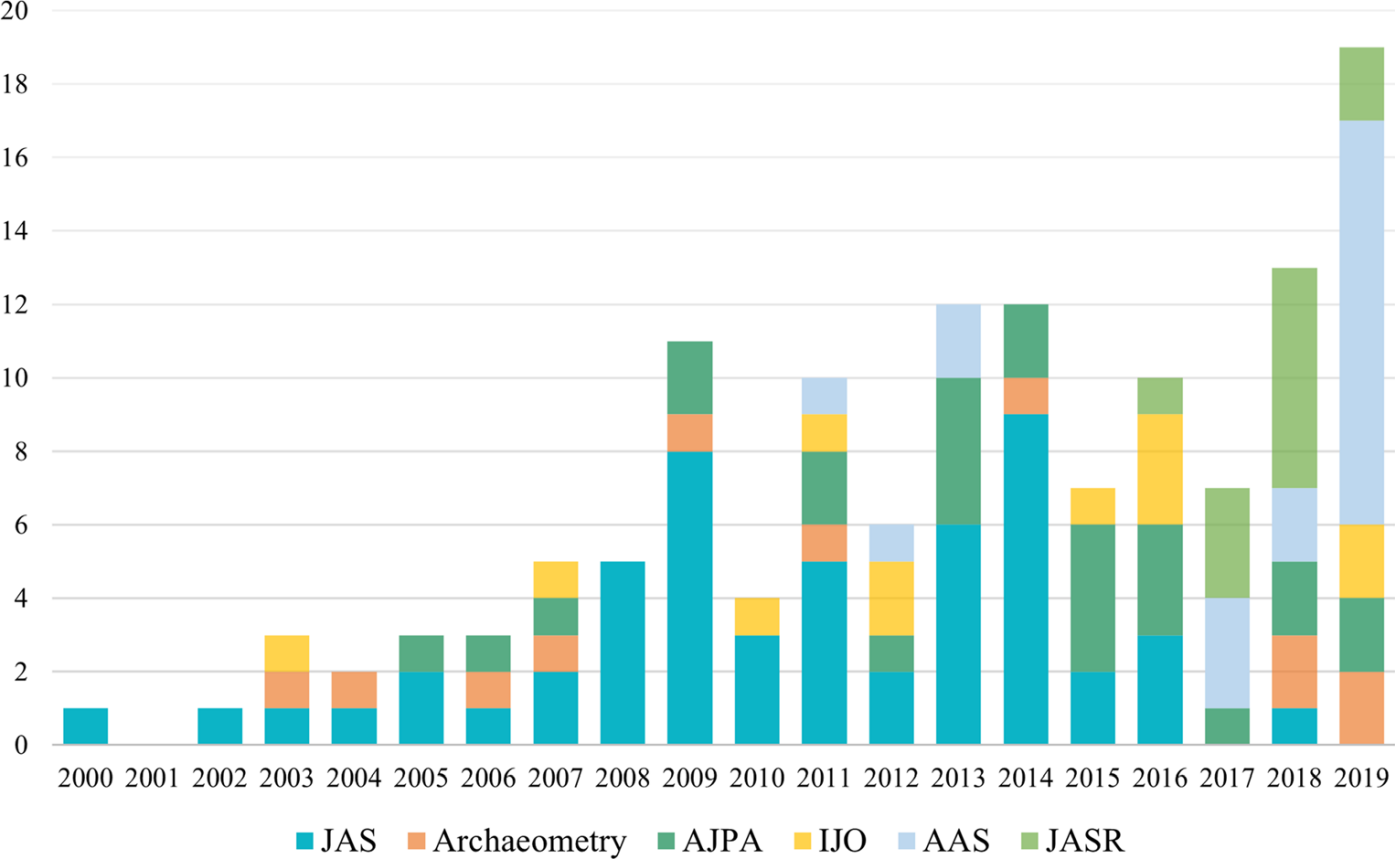
Review of recent (2000–2019) literature (*n* = 139) using ^87^Sr/8^6^Sr isotopes to analyse provenance, by year. Articles were systematically compiled from six relevant and impactful journals (*Journal of Archaeological Science* [*n* = 55], *Archaeometry* [*n* = 12], *American Journal of Physical Anthropology* [*n* = 26], *International Journal of Osteoarchaeology* [*n* = 13], *Archaeological and Anthropological Sciences* [*n* = 20], *Journal of Archaeological Science: Reports* [*n* = 13])

A recent systematic review of the bioarchaeological literature (September 2020) targeting six relevant and impactful archaeological and anthropological science journals (*Journal of Archaeological Science* [JAS], *Archaeometry*, *American Journal of Physical Anthropology* [AJPA], *International Journal of Osteoarchaeology* [IJO], *Archaeological Anthropological Sciences* [AAS], *Journal of Archaeological Science: Reports* [JASR]) demonstrates a temporal increase in the abundance of ^87^Sr/^86^Sr isotope analyses during the past 20 years, with some journal-specific publication trends (Fig. 1). While ^87^Sr/^86^Sr isotope analysis was abundant in the *Journal of Archaeological Science* between 2008 and 2014, publication of ^87^Sr/^86^Sr isotope analysis has sharply decreased in the last couple years. More recently established journals of *Archaeological and Anthropological Sciences* and *Journal of Archaeological Science: Reports* have published the highest number of ^87^Sr/^86^Sr isotope analysis studies since 2017. Given the results are presented as counts, they are subject to fluctuations in the publication rates of these journals; however, the goal of this systematic review was to assess the abundance of the ^87^Sr/^86^Sr literature. It should be noted that articles solely establishing a geological isoscape or synthesizing previously published data were excluded from the systematic review, as were articles focusing on modern skeletal samples, non-skeletal samples, and archaeological or fossil samples pre-dating 100,000 BP.

While it is common practice to sample local available fauna and botanicals to reconstruct environmental “baseline” values for ^87^Sr/^86^Sr isotope analysis of human remains, studies placing the primary or secondary research focus on zooarchaeological remains are rare (19.4%) relative to the abundant literature focusing solely on human remains (80.6%). Analysis of ^87^Sr/^86^Sr isotopes in faunal remains is valuable in and of itself and may provide evidence of animal husbandry practices, hunting practices, or animal trade networks*.* In some research contexts, fauna can serve as an accurate proxy for human movement. Analysing faunal as opposed to human skeletal remains has the added advantage of limiting the destructive sampling of human remains, a growing ethical concern in the discipline.

The majority of provenance studies surveyed (75.5%) simply analysed ^87^Sr/^86^Sr ratios in tooth enamel as opposed to ratios in bones or bones and tooth enamel, particularly if the research aim is simply to identify geological locals and migrants. While the earliest efforts to use ^87^Sr/^86^Sr ratios as an indicator of migration compared enamel (childhood) with bone (adult) values (Ericson 1985), the growing number of regional isoscapes available to researchers means that when it comes to distinguishing locals and non-locals, the bone no longer needs to act as a proxy for geographic region upon death. This, paired with the susceptibility of bones to diagenetic alteration and isotopic averaging effects of bones, has resulted in an increasingly sole focus on enamel. The bone may still be useful in some Sr isotope contexts; for example, recent research has shown that the process of cremation does not alter the ^87^Sr/^86^Sr isotope composition in skeletal remains and that the petrous portion of the temporal bone may therefore be a useful indicator of provenance for cremains (Harvig et al. 2014). The otic capsule of the petrous portion forms in utero to early infancy, is subject minimal remodelling, may be relatively more resistant to diagenesis than other bones, and often survives the process of cremation (Spence 1967; Harvig et al. 2014; Kontopoulos et al. 2019).

Pb has four stable and radiogenic isotopes (^204^Pb, ^206^Pb, ^207^Pb, ^208^Pb); the ratios of which have been successfully used as a Sr-esque indicator of provenance. A review of the recent literature reveals Pb isotopes are used far less often than ^87^Sr/^86^Sr isotopes (*n* = 14; Table 2). Pb isotopes are most commonly applied in multi-isotopic studies, in conjunction with the more commonly used isotopic tracers (^87^Sr/^86^Sr, δ^18^O), particularly in cases where ^87^Sr/^86^Sr or δ^18^O values lack geological specificity to pinpoint a specific region (e.g. overlapping isotopic values for multiple different regions) or are impacted by coastal ocean spray effects (Sharpe et al. 2019). Being far less applied than ^87^Sr/^86^Sr, this area of research is sometimes limited by the lack of geological Pb isotope data in different regions (Keller et al. 2016: 83). For early populations not associated with anthropogenic Pb, migration and mobility may be established via comparison of Pb isotopes in human remains with the geological bedrock signature (e.g. Valentine et al. 2008). However, for later populations, Pb pollution and frequent anthropogenic Pb exposure may overprint the “natural” geological Pb signature in bones and teeth (Sharpe et al. 2019), creating an isotopic average of multiple Pb mined ore sources collectively contributing to individual Pb burden. Pb isotope analysis of ore deposits from historically relevant mines is therefore crucial when undertaking mobility and migration analyses of populations from ancient and historical periods (Sharpe et al. 2019).

**Table 2:** Summary of recent (2000–2019) bioarchaeological Pb isotope research from six impactful archaeological science journals (JAS, *Archaeometry*, AJPA, IJO, AAS, JASR)

Study	Journal	Pb isotopes	Other isotopes?	Site or region	Time period
Bower et al. (2005)	IJO	^208^Pb/^204^Pb, ^207^Pb/^204^Pb, ^206^Pb/^204^Pb	No	Pueblo (CO, USA)	1879–1899 CE
Montgomery et al. (2005)	AJPA	^208^Pb/^206^Pb, ^207^Pb/^206^Pb, ^208^Pb/^204^Pb, ^207^Pb/^204^Pb ^206^Pb/^204^Pb	^87^Sr/^86^Sr	North Yorkshire (England)	5th–7th centuries CE
Valentine et al. (2008)	JAS	^208^Pb/^204^Pb, ^207^Pb/^204^Pb, ^206^Pb/^204^Pb	^87^Sr/^86^Sr	Sarawak (Malaysia)	3500–2300 BP
Turner et al. (2009)	JAS	^208^Pb/^204^Pb, ^207^Pb/^204^Pb, ^206^Pb/^204^Pb	^87^Sr/^86^Sr, δ^18^O	Macchu Picchu (Peru)	1438–1532 CE
Fitch et al. (2012)	IJO	^208^Pb/^206^Pb, ^207^Pb/^206^Pb	No	Grafton (IL, USA)	1832–1873 CE
Lamb et al. (2014)	JAS	^208^Pb/^206^Pb, ^207^Pb/^206^Pb, ^208^Pb/^204^Pb, ^207^Pb/^204^Pb ^206^Pb/^204^Pb	^87^Sr/^86^Sr, δ^18^O, δ^13^C, δ^15^N	Leicester (England)	1452–1485 CE
Shaw et al. (2016)	JAS	^208^Pb/^206^Pb, ^207^Pb/^206^Pb, ^208^Pb/^204^Pb, ^207^Pb/^204^Pb ^206^Pb/^204^Pb	^87^Sr/^86^Sr	London (England)	40–410 CE
Jones et al. (2017)	JASR	^207^Pb/^206^Pb, ^206^Pb/^207^Pb ^208^Pb/^204^Pb, ^207^Pb/^204^Pb, ^206^Pb/^204^Pb	^87^Sr/^86^Sr	Lower Illinois River Valley (IL, USA)	2100–1700 CE
Price et al. (2017a)	JAS	^208^Pb/^204^Pb, ^207^Pb/^204^Pb, ^206^Pb/^204^Pb	No	Sala (Sweden)	16th century CE
Price et al. (2017b)	JASR	^208^Pb/^204^Pb, ^207^Pb/^204^Pb, ^206^Pb/^204^Pb	^87^Sr/^86^Sr, δ^18^O, δ^13^C	Chaco Canyon (NM, USA)	10th–11th centuries CE
Evans et al. (2018)	*Archaeometry*	^208^Pb/^204^Pb, ^207^Pb/^204^Pb, ^206^Pb/^204^Pb	No	Weymouth (England)	970–1025 CE
Laffoon et al. (2019)	AJPA	^208^Pb/^206^Pb, ^207^Pb/^206^Pb, ^208^Pb/^204^Pb, ^206^Pb/^204^Pb	No	Barbados	17th–18th centuries CE
Price et al. (2019)	AAS	^208^Pb/^204^Pb, ^207^Pb/^204^Pb, ^206^Pb/^204^Pb	^87^Sr/^86^Sr, δ^18^O, δ^13^C	Tollense Valley (Germany)	1250 BCE
Tomczyk et al. (2019)	AAS	^208^Pb/^204^Pb, ^207^Pb/^204^Pb, ^206^Pb/^204^Pb	^87^Sr/^86^Sr, δ^18^O, δ^13^C, δ^15^N	Castillo de Huarmey (Peru)	600–1050 CE

### Trace elements and toxicity

In the twenty-first century, Pb has continued to be an important subject of study, with continued focus on past human Pb exploitation and health (e.g. González-Reimers et al. 2003; Nakashima et al. 2011; Montgomery et al. 2010; Schroeder et al. 2013; Millard et al. 2014; Pastorelli et al. 2014; Stipisic et al. 2014; Giffin et al. 2017; Laffoon et al. 2019; López-Costas et al. 2020; Rasmussen et al. 2020a; Scott et al. 2020). In doing so, a well-rounded history of human Pb use and exposure over the past several millennia has begun to emerge, as well as insights into the burden this may have on ancient and historical human health.

Environmental, social, and occupational determinants of human exposure to toxic elements beyond Pb have also been the subject of recent bioarchaeological study. Like Pb, arsenic (As) is an extremely toxic element that can result in skin pigmentations, liver diseases, gastrointestinal issues, central and peripheral neuropathies, cancers, and death (Hall 2002). In some arid areas of South America, freshwater reserves have naturally become contaminated with high levels of As (Pérez-Carrera and Cirelli 2010), causing some to hypothesize that certain ancestral populations in the area may have evolved, through positive selection, more efficient mechanisms of As metabolization to cope with this environmental stressor (Apata et al. 2017). High levels of As in skeletal remains have consistently been found. For example, Swift et al. (2015) studied As concentrations of skeletal (bone and dental) remains in the Atacama Desert, Chile, during the Late Chinchorro to Inca periods (3867–474 BP), inferring that over several millennia, approximately one third of the population would have likely suffered from chronic arseniasis (> 1 ppm As) as a result of the contaminated drinking water in this region.

Mercury (Hg) toxicity has also been the subject of extensive bioarchaeological study over the past two decades; like Pb, Hg compounds like cinnabar have been widely used in various ancient and historical contexts. Studies have inferred the health impacts of both Hg atmospheric pollution and regular contact with anthropogenic sources of Hg on past populations (Ávila et al. 2014; Emslie et al. 2019; Álvarez-Fernández et al. 2020; López-Costas et al. 2020). For example, Emslie et al. (2019) analysed biogenic Hg exposure in numerous Neolithic to Bronze Age Iberian populations that were known for their extensive use of cinnabar-based pigments, finding that interestingly humeri disproportionately contained greater concentrations of Hg, which the authors speculated could be caused by greater blood flow to this skeletal region and/or higher, load-induced remodelling rates in the humerus. Others have also examined anthropogenic exposure to other metals, such as Cu. For example, Rasmussen et al. (2020b) tracked Cu exposure across pre- and post-medieval Denmark, finding that urban individuals experienced significantly higher copper exposure than their rural counterparts, which they attribute to variation in social status and the availability of metallic household goods. While having critical health repercussions for individuals, metal pollution can, on the flipside, introduce higher levels of toxic TEs into regional environmental circulation, so diagenetic contamination may present an even more pervasive concern for studies geared toward this topic. Where possible, researchers should apply multiple methods of analysis to ascertain the extent of diagenetic alteration before making biogenic inferences.

Another interesting area of research is investigating the extent that the skeletal toxic TE burden among past individuals stems from medicinal and care practices of past populations. Both As and Hg have historically been used as medicine by health practitioners from various populations (Goldwater 1972; Jolliffe 1993); some bioarchaeological studies have consequently investigated cases of As and Hg toxicity potentially resulting from medicinal treatments (Tucker 2007; Rasmussen et al. 2008; Kępa et al. 2012; Swanston et al. 2015; Walser III et al. 2018; Dabrowski et al. 2019). Comparatively high levels (16.17 ± 0.58 μg/g) of skeletal As were discovered in an individual from Wroclaw, Poland (late sixteenth to mid-eighteenth centuries CE) with skeletal signs of syphilis, indicating As was used as a medicine (Dabrowski et al. 2019). Similarly, high levels of mercury (Hg) were found among Danish monks interred at the Cistercian Abbey cemetery at Øm but not at the Franciscan Friary of Svenborg (Rasmussen et al. 2008). This pattern was attributed to Cistercian monks treating leprosy and syphilis patients with Hg-containing medicine, or alternatively, from using Hg ink in the Abbey’s scriptorium (Rasmussen et al. 2008). While these studies reporting high concentrations of toxic elements may be indicative of chronic treatments, short-term treatments with these toxic compounds would perhaps not be detectable in the bioarchaeological record, given that TEs are limited to incorporation within actively forming and mineralizing regions of the skeleton.

### Multi-element analyses

Despite the 1990s critiques, small pockets of scholars continue to use TEs for palaeodietary inferences. Some of these studies (e.g. González-Reimers et al. Szostek et al. 2003; Busetto et al. 2008; Corti et al. 2013; Ïzci et al. 2013; Bianchi et al. 2017; Guede et al. 2017; Bocca et al. 2018; Rasmussen et al. 2020a) have continued to suggest that elements, such as Ba, Cu, Fe, Mg, Mn, molybdenum (Mo), nickel (Ni), Sr, and Zn, among others, can be used to make direct inferences about palaeodiet. In doing so, these studies often rely upon the correlational literature from the 1980s to advocate for the use of TE concentrations and ratios to deduce the relative contributions of different dietary constituents, such as terrestrial animal and marine proteins versus plants. Often, these studies further subdivided these general dietary categories, using TE concentrations to infer consumption of specific foods such as cereals and breads, vegetables, nuts, red meats, and fish. These recent studies fail to account for the complex fractionation and regulatory mechanisms of essential TEs as outlined in the physiological literature and often do not satisfactorily account for the effects of diagenesis, attributing variation in TE concentrations to palaeodiet insights as opposed to diagenesis (Lugli and Cipriani 2017, 2018). It is not clear why these studies prevail in the literature; perhaps there are temptations to draw palaeodietary conclusions when data seems to align with these old notions of TEs and diet, or perhaps in light of research and publication demands, due diligence in surveying the full extent of the literature has not been sufficiently undertaken. While critics have retroactively commented on the perseverance of these studies following their publication, it is also both the responsibility of researchers to make conservative judgements on palaeodiet if using TE concentrations, and the responsibility of reviewers and journal editors to carefully consider the legitimacy of palaeodietary claims and the cited literature on which the claims stand.

Based on their critiques of surviving TE palaeodietary research, Lugli and Cipriani (2018) recommend that for TEs to be properly applied toward palaeodiet analysis, several criteria must be met. First, diagenetic alteration of skeletal remains must be properly accounted for; second, essential TEs should be disregarded as palaeodietary indicators; and third, proper ecological baseline analyses must be conducted before using Sr/Ca or Ba/Ca ratios for palaeodietary inference. Some studies have successfully addressed these criteria and employed TEs in conjunction with other lines of evidence to make more careful and comprehensive palaeodietary inferences (Arnay-de-la-Rosa et al. 2011; Lazzati et al. 2016; Lugli et al. 2017; Walser III et al. 2020). For example, Walser et al. (2020) used TEs (Ba, Pb, Sr, Zn) to bolster inferences on geographic provenance, diet, and health within a sixteenth-century Icelandic population and cautiously contextualized essential TE data against the known limitations and physiological literature.

Interestingly, in a recent 2016 study on the medieval Italian site of Caravete, Lazzati and colleagues analysed the population’s diet by through multicollector (MC)-ICP-MS analysis of 22 TEs in dental calculus (tartar) samples, supplemented with optical and scanning electron microscopy of the phytoliths within calculus. Based on both the TE and phytolith evidence, they hypothesized that this population primarily consumed fish protein and carbohydrates belonging to dicot and monocot (specifically Poaceae, or grasses) plant families. This novel approach to using dental calculus for TE palaeodietary reconstruction potentially overcomes the issue of regulatory effects of metabolism on TEs in bones and teeth, because calculus deposits on teeth secondarily. These data echo earlier promising results from the 1990s on the utility of calculi (Capasso et al. 1995). However, this particular study was perhaps premature; before this approach should be further applied, additional research should be conducted on the relationship between diet and element values in calculus, as well as the influence of diagenesis and microbial activity on the elemental composition of calculus.

While Sr isotope analysis has dominated twenty-first-century TE studies, Sr/Ca ratios have occasionally continued to be used in weaning and diet studies. Because breastmilk is depleted in Sr relative to Ca, but weaning foods contain more Sr, Sr/Ca ratios have utility in reconstructing breastfeeding and weaning patterns in past populations. By comparing Sr/Ca ratios against estimated age-at-death for juvenile individuals, Mays (2003) reported that the average weaning age among the medieval Wharram Percy population was between one and two years. From a dietary perspective, Sr/Ca and Ba/Ca ratios have continued to be successfully applied to cautiously interpret relative dietary contributions (e.g. plant, marine foods; Lugli et al. 2017). For example, Sr/Ca ratios were used in conjunction with stable C, N, and sulphur (S) isotope data to analyse the diet of gladiators and civilians from a Roman-era cemetery in Turkey (Lösch et al. 2014). The authors reported that the highly elevated Sr/Ca ratios in gladiator bone relative to civilians is likely indicative of the gladiators’ frequent consumption of a plant ash beverage used as a post-fight remedy (Lösch et al. 2014).

## Resurgence of trace element analysis? Techniques with growing momentum

### Micro-sampling techniques

The “biostratified nature” of skeletal remains means that they contain a record of growth, modelling, and drift events (Maggiano et al. 2016: 191) and, by extension, TE exposure dynamics. However, because most solution-based chemical analytical methods require skeletal sample amounts in the order of milligrams to grams (Castro et al. 2010), these conventional methods often produce element concentrations and ratios reflecting a chemical average of up to several years. Within the field of archaeological TE analysis, approaches to counteract this limitation and gain temporal specificity have included comparing the chemical composition of different skeletal tissues (e.g. bone vs enamel, enamel vs dentin; Ericson 1985; Price et al. 2000; Bower et al. 2005; Stojanowski and Knudson 2011; Wright et al. 2019), sampling from multiple teeth developing at different stages (Schweissing and Grupe 2003; Turner et al. 2009; Stojanowski and Knudson 2011; Knipper et al. 2016), or comparing trabecular and cortical bone values from multiple bones with different turnover rates (Skytte and Rasmussen 2013; Rasmussen et al. 2013, 2016). Stable isotope studies have also sampled from different bone density fractions (Bell et al. 2001) and compared samples from the developed diaphysis and developing metaphysis of a bone (Waters-Rist et al. 2011)—approaches which could feasibly be applied to archaeological TE analysis.

Micro-sampling techniques (e.g. acid-leaching, micro-sectioning, micro-milling, micro-drilling, laser ablation) are a means of accessing bioarchaeological TE data with great specificity, involving the extraction of small samples that potentially correspond to discrete microstructures in teeth and bone for elemental analysis (Outridge et al. 1995). Early efforts to assess variation in the distribution of TEs across dental tissues date back to at least the mid-twentieth century, where researchers analysed sequential sections or etched and acid-treated layers of enamel (e.g. Steadman et al. 1958; Weatherell and Hargreaves 1965). Precision in micro-sampling methods (e.g. micro-milling, micro-drilling, micro-sectioning) has continued to improve through the decades.

Micro-sampling from distinct bone and dental microstructures further provides the potential for assessing an individual’s “chemical life history” (Skytte and Rasmussen 2013) with higher temporal resolution and combatting diagenesis. This approach is also advantageous to the field as a whole by minimizing the extent of destruction to precious skeletal material in an era of growing concern over the ethics of destructive methods (Stadlbauer et al. 2007). In this vein, Dolphin et al. (2016) proposed the possibility of strategically grouping together multiple destructive analyses (e.g. TE analysis, stable isotope analysis, histology, FTIR) within a single tooth to gain insights into multiple lines of osteobiographical evidence. The minute quantities required for element analysis made possible by micro-sampling eliminates the need to sample multiple teeth for different analyses while also reserving sufficient dental material for future research.

Unlike bones, which continually remodel, the main constituents of teeth (dentin, enamel, cementum) form incrementally and thus have the potential to provide a timeline of element exposure. Incremental sequential sectioning of enamel has been successfully applied to zooarchaeological specimens such as to infer seasonality, husbandry practices, and animal trade networks through examining short-term shifts in ^87^Sr/^86^Sr isotopic composition (Balasse et al. 2002; Bendrey et al. 2009; Viner et al. 2010; Fisher and Valentine 2013; Chase et al. 2014; Price et al. 2017b; Evans et al. 2019). This approach of sequentially sampling TEs within dental increments for ^87^Sr/^86^Sr isotope analysis is less commonly seen in archaeological studies on humans, with rare exceptions (e.g. Nugent 2019).

Further spatial (and consequently, temporal) sensitivity can be attained with laser ablation (multicollector)-inductively coupled plasma-mass spectrometry (LA-[MC-]ICP-MS), in which a laser with a spot size of as little as a few microns extracts a micro-sample from a bone or tooth section. Consequently, element concentrations and isotopic ratios corresponding to discrete bone and dental microstructures can be sampled with even greater spatial resolution. This has been successfully applied to examine concentrations, elemental ratios, and isotopic ratios in both archaeological bones (e.g. Kępa et al. 2012; Scharlotta et al. 2013; Dudgeon et al. 2016) and teeth (e.g. Humphrey et al. 2007; Simonetti et al. 2007; Richards et al. 2008; Cucina et al. 2011; Dudgeon et al. 2016; Mays et al. 2018; Meijer et al. 2019).

LA-(MC-)ICP-MS may provide a means to recover biogenic element concentrations from diagenetically contaminated samples. For data, to study hunter–gather mobility, Scharlotta et al. (2013) used LA-MC-ICP-MS to analyse intra-osteon ^87^Sr/^86^Sr isotopic ratios in bone samples from the Khuzhir-Nuge Xiv cemetery (c. 4650–4250 years BP) of the Cis-Baikal region, Siberia, wherein it was suspected that the remains were partially diagenetically altered. The authors visually identified diagenetically altered regions of each sample and ablated non-overlapping lines (each line representing a timespan within one month and one year of life) within osteons either identified as “intact” or “diagenetic.” In doing so, the authors both recovered biogenic Sr information and characterized diagenetic Sr chemical profiles. Some studies (e.g. Richards et al. 2008; Mays et al. 2018; Meijer et al. 2019) have also successfully employed LA-(MC-)ICP-MS to study intra-enamel ^87^Sr/^86^Sr variability and, in doing so, gained insights into human mobility and seasonal migrations, phenomena which are often more difficult to detect isotopically from bulk enamel samples than outright childhood migration. These novel studies demonstrate the utility of micro-sampling in the face of common limitations in the chemical analysis of skeletal remains—specifically, reducing destructive sampling, improving temporal resolution to gain insights into individual “chemical life histories”, and combatting the potential impacts of diagenesis on biogenic signatures.

### Element mapping

Similarly, element mapping can aid in differentiating the nature of element uptake in skeletal remains by improving temporal resolution or distinguishing biogenic from diagenetic exposure. In the bone, osteonal microstructures can be relatively “dated”; interstitial osteon fragments represent mature bone that survived remodelling events, whereas osteons are relatively newer formations, and younger osteons may be hypomineralized (having not completed secondary mineralization) and/or have a larger Haversian canal relative to mature osteons. In teeth, the incrementally forming layers of enamel, dentin, and cementum can be delineated microscopically. Because TEs are only incorporated biogenically into actively forming and mineralizing bone and dental structures, a visual distribution of TEs in relation to structures represents a record of lifetime exposure. Additionally, it would be expected that biogenic and diagenetic TE distributions would vary due to their different mechanisms of uptake. Element mapping of bone and tooth samples can be achieved through techniques such as synchrotron radiation X-ray fluorescence imaging (SR-XFI; Carvalho et al. 2004; Martin et al. 2004, 2007a, b; Wittmers et al. 2008; Swanston et al. 2012, 2015, 2018; Dolphin et al. 2013; Choudhury et al. 2016, 2017), LA-(MC-)ICP-MS (Humphrey et al. 2008; Galinová et al. 2013; Dudgeon et al. 2016; Willmes et al. 2016; Maurer et al. 2019; Rasmussen et al. 2019), or proton-induced X-ray emission (PIXE; Coote et al. 1982; Buoso et al. 1992; Elliott and Grime 1993).

**Fig. 2:**
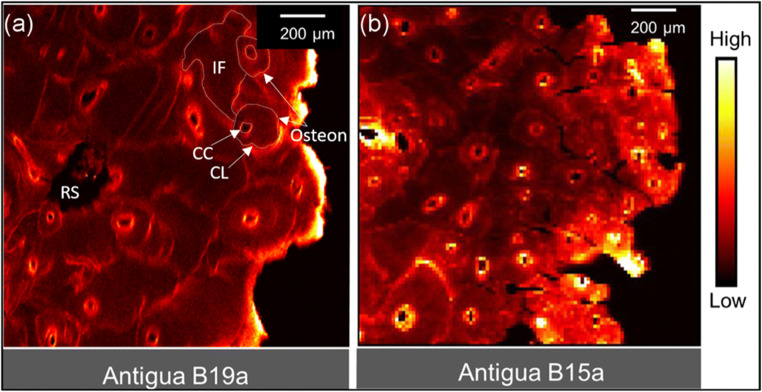
**a**, **b** SR-XFI elemental map of Pb from cortical bone samples from two British Royal Navy personnel from colonial Antigua. The intensity of Pb varies in accordance with bone microarchitecture, wherein cement lines (CL) and central canals (CC) of osteons are enriched. Interstitial fragments (IF) of former osteons and primary lamellae are similarly enriched. Resorption spaces (RS) represent active regions of bone remodelling. This naval population experienced high levels of sustained Pb exposure (reproduced and modified from Swanston et al. 2018, PLoS One, under Creative Commons Attribution (CC BY) license)

Compared with most solution-based analytical techniques, SR-XFI is a relatively non-destructive technique that uses a very focused X-ray microbeam to two-dimensionally map multiple trace elements within a given sample. In their early exploratory study, Martin et al. (2007a) used SR-XFI to study the distribution of Br, Ca, Pb, and Zn in ancient Peruvian bone and teeth samples, finding that Zn and Pb disproportionately accumulated in the cementum and periosteal surface of bone, while Br accumulated in Ca-deficient regions of bone and the Haversian canal surfaces of bone. As it was hypothesized that navy personnel in colonial Antigua experienced Pb toxicity due to leaded rum distillation equipment, Swanston et al. (2012) used SR-XFI to generate a two-dimensional fluorescence map of the microdistribution of Pb in bone samples from a British Royal Navy hospital cemetery; the spatial resolution was later improved upon with the addition of confocal optics (Swanston et al. 2018; Fig. 2). Pb was unevenly distributed across each bone sample, though focused in the cement lines, Haversian canals, and interstitial fragments of osteons, which provided evidence for biogenic Pb exposure sustained over a long period of time. The same approach was later used to map the distribution of biogenic Hg in a bone sample from an individual with an unusually high bone Hg concentration (94.6 μg/g; Swanston et al. 2015). The biogenic nature of Hg was confirmed by employing synchrotron radiation X-ray absorption spectroscopy (XAS), a synchrotron technique which can identify the organic, inorganic, or elemental forms of a given element, some of which are more likely to be biogenic or diagenetic in origin (Swanston et al. 2015).

SR-XFI of teeth has similarly provided insights into metabolism, diet, and the nature of element exposure. Dean et al. (2018, 2019) used SR-XFI to map variation of Ca, Sr, and Zn in fossil primate and modern human teeth, finding that Sr varies pre- and postnatally due to differences in serum levels, and that cementum increments and the neonatal line were particularly rich in Zn. Studies have also used SR-XFI to spatially examine Br and Pb in cementum in order to elucidate marine dietary components and the timing of element exposure, respectively (Dolphin et al. 2013; Swanston et al. 2018).

**Fig. 3:**
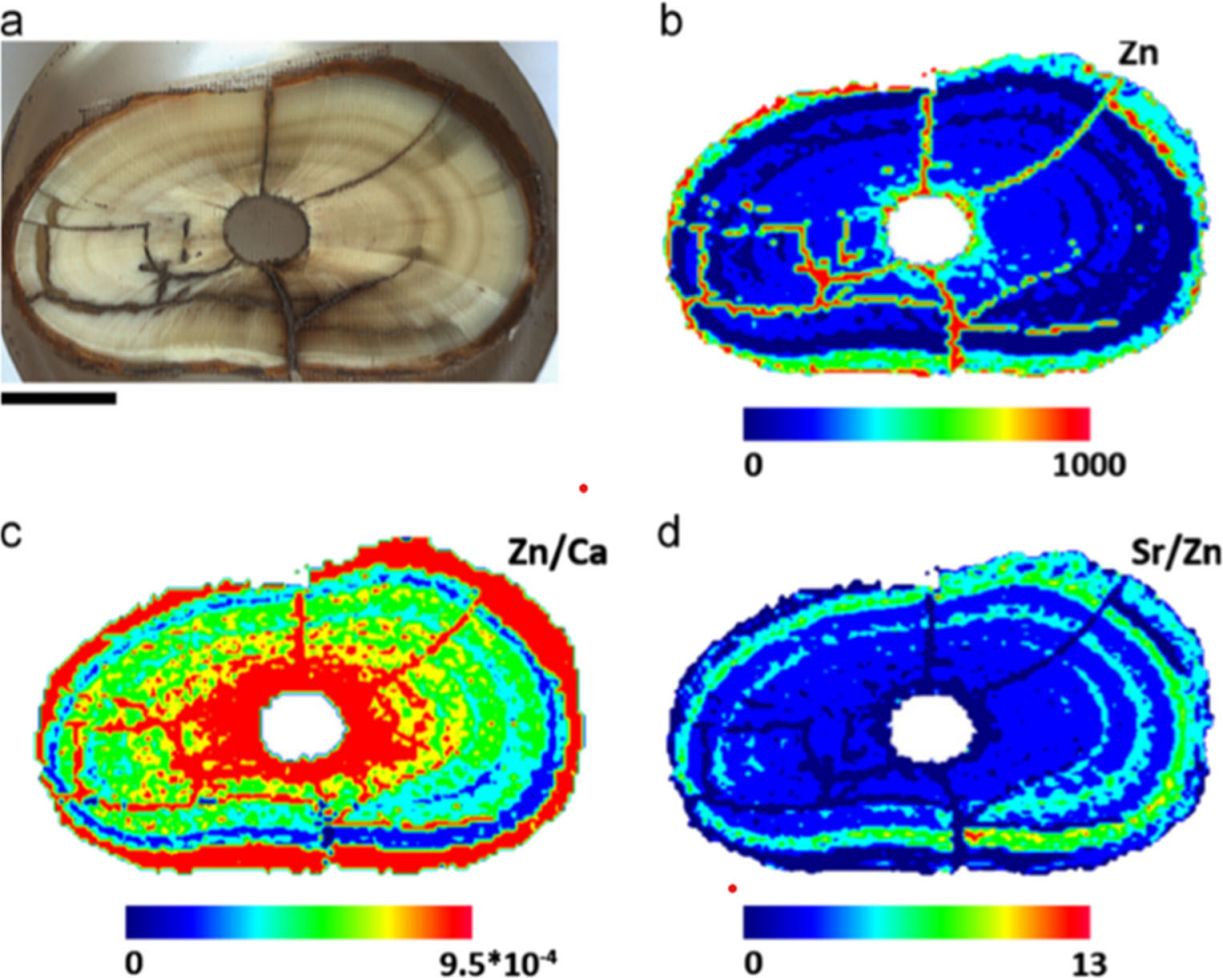
Within the brown bear (Ursus arctos) canine tooth root pictured in (**a**), element distribution maps of Zn (**b**), Zn/Ca ratios (**c**), and Sr/Zn ratios (**d**) were compiled using LA-ICP-MS. Zn, Sr, and Ca distributions in dental increments were used to infer seasonal migration, feeding, and hibernation patterns (originally published in Galinová et al. 2013 and reproduced with permission from Talanta)

If numerous ablation spots are taken from a bone or tooth sample, LA-ICP-MS can also be used to spatially map the distribution of elements. Galinová et al. (2013) used LA-ICP-MS to map Ba, Ca, P, Sr, and Zn in a tooth root from a brown bear fossil specimen. In doing so, they reconstructed seasonal migration and feeding trends, finding an elevation in the Sr/Zn ratio in winter season bands corresponding to hibernation (Fig. 3). LA-ICP-MS mapping can also be used to identify biogenic and diagenetic TE uptake in bones and teeth. For example, Rasmussen et al. (2019) used LA-ICP-MS to map diagenesis of Ba, Cu, Fe, Mn, Pb, and Sr in both modern and archaeological Danish bone, demonstrating diagenetic enrichment strictly on the outer surfaces of bone and within Haversian canal networks and Willmes et al. (2016) successfully recovered biogenic Sr data from diagenetically altered human teeth using LA-ICP-MS mapping.

SR-XFI is relatively less destructive than LA-ICP-MS, but quantification of elements is more refined with LA-ICP-MS. Employing confocal SR-XFI and LA-ICP-MS requires a sufficiently thin sampling “thickness” so that a high degree of certainty of co-localization between the elements of interest and bioarchitecture of the bone/teeth is achieved. This co-localization certainty is somewhat greater for confocal SR-XFI given the ability to achieve typically smaller beam focus.

#### Modern studies using micro-sampling and elemental mapping methods

Whether intentionally or not, biomedical and biological anthropological researchers studying skeletal samples from modern individuals have, in part, helped address Joseph Ezzo’s (1994) call for empirical physiological and biomedical literature on homoeostatically controlled TEs, such as Zn. Researchers using microsampling and element mapping techniques on modern samples often have the advantage of known demographic and life history variables for populations, allowing more direct and biocultural inferences about TE dynamics in bones and teeth to be made. These inferences may in turn be successfully extrapolated to TE phenomena in the bioarchaeological record.

TE research on modern human populations using micro-sampling techniques (e.g. Goodman et al. 2003; Dolphin et al. 2005; Dolphin and Goodman 2009; Humphrey et al. 2007; Castro et al. 2010; Farell et al. 2013) may help to provide insights into interpreting TE patterns observed in skeletal remains from past populations. Such studies have examined TE deposition patterns within discrete skeletal structures in relation to factors such as development, nutrition, physiological stress, disease, and environmental pollution. For example, Dolphin and Goodman (2009) used LA-ICP-MS to examine micro-variation in Zn accumulation in modern deciduous enamel from children in Solís, Mexico, particularly in relation to social status, maternal nutrition (e.g. maize, phytate, Ca consumption), and adaptive Zn absorption strategies and their impacts on the Zn metabolism of children. LA-ICP-MS has also been used to measure fluctuations in Sr/Ca ratios throughout enamel layers in deciduous teeth to reconstruct weaning histories among modern children with known feeding strategies (Humphrey et al. 2007).

Element mapping of bone and dental samples from modern humans has also provided valuable insights into human TE metabolism and interactions between TEs and diet or disease. Such research has provided insights into biogenic “baselines” and metabolic patterns of TE accumulation in bone and dental tissues (Kang et al. 2004; Zoeger et al. 2005, 2008; de Souza-Guerra et al. 2010, 2014; Pemmer et al. 2013; Wang et al. 2017). Use of SR-XFI to map the disproportionate distribution of TEs such as Pb and Zn in modern bone with respect to diseases such as osteoarthritis and osteosarcomas (Zoeger et al. 2008; Rauwolf et al. 2017) may also aid in palaeopathological interpretations, potentially including cases where the diseases were still in early stages at death. Overall, research in this area has produced several valuable takeaways for the bioarchaeological interpretation of TE variation within specific developmental junctures.

### Non-traditional stable isotopes

Conventionally, the “traditional” non-radiogenic stable isotopes of carbon (δ^13^C), nitrogen (δ^15^N), sulphur (δ^34^S), and oxygen (δ^18^O), and radiogenic stable isotopes of strontium (^87^Sr/^86^Sr) and lead (^206^Pb/^204^Pb) have been used to establish palaeodiet, migration patterns, weaning, and social status in past populations (Burton 2008; Katzenberg and Waters-Rist 2019). However, innovative developments in multi-collector inductively coupled plasma-mass spectrometry (MC-ICP-MS) and thermal ionization mass spectrometry (TIMS) have allowed for the differentiation of isotopes from heavier elements, such as copper (δ^65^Cu), iron (δ^56^Fe, δ^57^Fe), magnesium (δ^25^Mg, δ^26^Mg), mercury (δ^202^Hg, δ^199^Hg), zinc (δ^66^Zn, δ^67^Zn, δ^68^Zn), and non-radiogenic strontium (δ^88^Sr; Jaouen and Pons 2017). Non-traditional trace isotope systems may permit the field of TE analysis to revisit palaeodietary reconstruction and open new doors to the aspects of migration and provenance, metabolism, deficiency, sex estimation, and life history.

As discussed above, a key critique faced by the field of TE analysis was the use of elements (e.g. essential elements) inappropriate for palaeodiet reconstruction. Variation in element concentrations of consumed plants do not correspond to element concentrations of bone if said elements are regulated by homoeostatic control mechanisms (e.g. Cu, Fe, Mg, Zn), except in cases of extremely deficiency or toxicity. Still in its infancy, the analysis of “non-traditional” stable isotopes (Jaouen and Pons 2017) is a potential means of addressing this limitation because biogenic bone isotopic ratios are more likely to reflect dietary factors. Utilizing “non-traditional” isotopes for palaeodiet construction has the added benefit of using hydroxyapatite-based elements in cases where the organic collagen or dentin preservation is poor, such as in reconstructing hominin diet (Jaouen 2018).

Several studies have provided insights into the ecological dynamics of several trace metal isotopes, which have implications for palaeodietary reconstruction. For example, Jaouen et al. (2013) studied the fractionation of Cu, Fe, and Zn isotopes in mammalian food webs, reporting that plant consumption results in the preferential absorption of heavier Zn isotopes. Jaouen et al. (2016a, 2016b) found a marked trophic level effect for δ^66^Zn values in terrestrial and marine mammals in the Turkana Basin and Canadian Arctic, respectively. With regard to Fe isotopes, plant metabolisms vary with respect to light versus heavy Fe isotopes and employ one of two uptake strategies that may be useful in differentiating specific plants in one’s diet (Guelke and von Blanckenburg 2007). Von Blanckenburg et al. (2013) established discrete δ^56^Fe isotopic ranges for vegetables, grain crops, and animal products. These studies and their palaeodietary implications are somewhat reminiscent of attempts from decades ago. However, unlike element concentrations subjected to a host of regulatory processes, a body’s isotopic ratios are impacted by both isotopic ratios in food and metabolic fractionation mechanisms; therefore, by comparing the isotopic signature of an element in remains against the isotopic signatures of local bioavailable foods and known human isotope fractionation processes, it may be possible to gain interesting insights into palaeodiet.

Some scholars have proposed applications of certain “non-traditional” stable isotopes to bioarchaeology beyond the realm of palaeodiet reconstruction. Similar to Sr and Pb, skeletal isotopic variation in Mg and Zn related to geographic isotopic variation has been found (Martin et al. 2014; Melin et al. 2014; Jaouen et al. 2016a, as cited in Jaouen and Pons 2017), demonstrating the potential utility of these isotopes for establishing provenance. In cases where ^87^Sr/^86^Sr, ^208,207,206^Pb/^207,206,204^Pb, or δ^18^O isotopes do not provide clear evidence for migration or mobility (e.g. non-specificity due to overlapping values in multiple regions), using additional isotope systems may be advantageous. Hg stable isotopes (δ^202^Hg, δ^199^Hg) in human remains have been used to trace anthropogenic exposure to cinnabar deposits and consequently infer trade routes and mobility ranges (Emslie et al. 2015). Before these new isotope systems can be widely used for this purpose, however, further research into the particulars of isotopic fractionation and in-depth reconstructions of regional isoscapes need to take place.

Assessing the isotopes of essential elements under homoeostatic control may also provide insight into cases of nutritional deficiency or metabolic dysregulation (Jaouen and Pons 2017). For example, Mg, Fe, Cu, and Zn are essential TEs that perform critical metabolic and physiological functions. Due to intrinsic homoeostatic mechanisms, nutritional deficiency of such TEs may not be immediately evident by analysis of bulk skeletal levels, but Jaouen and Pons (2017) suggest the unregulated isotopic ratios may provide insights into TE deficiency or TE regulation pathologies (e.g. stemming from hemochromatosis, Fe deficiency, Wilson disease, cancer, bone mineral [im]balance).

Non-traditional isotopes may also aid in skeletal sex (and age) estimation. Jaouen et al. (2012) found significant sex differences in ^56^Fe/^54^Fe and ^65^Cu/^63^Cu isotopic ratios in bone samples from a seventeenth- to eighteenth-century French population, reflecting the differences in the isotopic composition of blood between males and females. They suggested that, with further research on animal models and human populations, these findings could potentially be translated into an alternative approach for sex estimation of skeletal remains. The authors proposed that this sex-specific variation in bone likely reflects a metabolic phenomenon in blood reported by Walczyk and von Blanckenburg (2002), in which ^56^Fe and ^65^Cu are depleted among females when compared to males. Sex-specific variation has been confirmed in follow-up studies, which show that the ^56^Fe/^54^Fe and ^65^Cu/^63^Cu isotopic composition of blood shifts post-menopause, demonstrating the potential utility of ^56^Fe/^54^Fe and ^65^Cu/^63^Cu for estimating both sex for premenopausal females vs males and age of menopause in females (Jaouen and Balter 2014). As Jaouen and colleagues point out, further studies need to be undertaken before this method is widely applied archaeologically. The fractionation dynamics of Fe and Cu isotopes from blood to bone need to be better understood, as does ^56^Fe/^54^Fe and ^65^Cu/^63^Cu isotopic variation among populations exhibiting a broader range of reproductive histories (different mean ages at menarche, interbirth spacing, breastfeeding duration, contraceptive methods).

While still in its infancy, the emerging field of “non-traditional” stable isotopes is a promising direction for bioarchaeological TE analysis that may re-open the door to TE analysis of chemicals previously deemed unsuitable. Hopefully, bioarchaeologists will be influenced by the cautionary tales of overzealous multi-element analyses of the 1980s, however, and continue to carefully research these isotopes in experimental and ecological contexts before widely applying them to the bioarchaeological record.

## Conclusions

This review has provided a historical overview of the field of bioarchaeological TE analysis from the past six decades and analysed the current state of the field by investigating common trends and innovative approaches. Mid-twentieth-century efforts to characterize human bone chemistry and interpret diet from Sr in skeletal remains culminated in the widespread use of a multiplicity of elements for bioarchaeological palaeodietary interpretations during the 1980s. The early optimism for several TEs to carry significance about past human lifeways was called into question, however, by critics who argued that many scholars failed to take into account the impacts of diagenesis and the complexities of geological, ecological, and metabolic TE dynamics.

This period of critiques resulted in the relative abandonment of TEs for palaeodietary reconstruction, except in cases of extreme deficiency or toxicity, though the use of TE isotopes for reconstructing mobility and geological origins has proved fruitful in the twenty-first century. Analyses of elemental exposure to toxic (mostly non-essential) TEs has also remained a constant avenue of bioarchaeological exploration, providing new understandings into the interplay of human activities and health in the past. Some small pockets of studies have continued to rely on surviving naïve misconceptions in the literature that advocate for direct palaeodietary inferences from TEs, including regulated essential TEs. It is not clear why this literature prevails because at present, contemporary physiological and metabolic literature does not support such inferences.

Developments in micro-sampling techniques, element mapping methods, and “non-traditional” stable isotope analysis may help combat previous critiques of the field of TE analysis and provide a means to revisit earlier thinking. Micro-sampling and element mapping of bone and teeth present potential means to account for diagenesis, recover biogenic information, and provide better temporal specificity into the “chemical life histories” of individuals, while often reducing the extent of destructive sampling. These areas of research are aided by a multiplicity of controlled, modern studies that have investigated TE accumulation in skeletal tissues in relation to biocultural phenomena. Typically unaffected by homoeostatic regulation, “non-traditional” isotopes may re-open the door to the study of essential and regulated elements previously deemed unsuitable by critics, while also providing a new means of studying migration, provenance, life history, metabolism, and disease among past populations. The most effective bioarchaeological TE research from the past two decades has been carefully situated within both population-specific biocultural contexts and the existing array of physiological, biochemical, and anthropological literature. This area of research would benefit from continued investigation in, and engagement with, modern and experimental research and the holistic utilization of multi-faceted approaches to assessing diagenesis. It is our view that when employed cautiously and knowledgeably, TE analysis has potential to be a valuable re-addition to the twenty-first-century bioarchaeologist’s toolkit.

## Supplementary Information

ESM 1 (XLSX 28 kb)

## Data Availability

Not applicable
